# Exploring the mRNA expression level of *RELN* in peripheral blood of schizophrenia patients before and after antipsychotic treatment

**DOI:** 10.1186/s41065-020-00158-6

**Published:** 2020-11-06

**Authors:** Jiajun Yin, Yana Lu, Shui Yu, Zhanzhan Dai, Fuquan Zhang, Jianmin Yuan

**Affiliations:** 1 Brain Science Basic Laboratory, The Affiliated Wuxi Mental Health Center with Nanjing Medical University, 156 Qianrong Road, Wuxi, 214151 Jiangsu Province P.R. China; 2 Department of Psychiatry, The Affiliated Wuxi Mental Health Center with Nanjing Medical University, Wuxi, Jiangsu Province China; 3 Department of Psychiatry, The Affiliated Brain Hospital of Nanjing Medical University, 264 Guangzhou Road, Nanjing, 210029 Jiangsu Province P.R. China

**Keywords:** *RELN*, SCZ, Antipsychotic treatment

## Abstract

**Background:**

The Reelin *(RELN)* gene encodes the protein reelin, which is a large extracellular matrix glycoprotein that plays a key role in brain development. Additionally, this protein may be involved in memory formation, neurotransmission, and synaptic plasticity, which have been shown to be disrupted in schizophrenia (SCZ). A decreasing trend in the expression of *RELN* mRNA in the brain and peripheral blood of SCZ patients has been observed. There is a need to determine whether changes in *RELN* mRNA expression in SCZ patients are the result of long-term antipsychotic treatment rather than the etiological characteristics of schizophrenia. The expression levels of *RELN* mRNA in the peripheral blood of 48 healthy controls and 30 SCZ patients before and after 12-weeks of treatment were measured using quantitative real-time PCR.

**Results:**

The expression levels of *RELN* mRNA in the SCZ group were significantly lower than that of healthy controls; however, after 12-weeks of antipsychotic treatment, *RELN* mRNA levels were significantly increased in the SCZ group.

**Conclusion:**

The up-regulation of *RELN* mRNA expression was current in SCZ patients after antipsychotic treatment, suggesting that the changes in *RELN* mRNA expression were related to the effect of the antipsychotic treatment.

## Background

Schizophrenia (SCZ) is a common and severe mental disorder, with a lifetime prevalence estimate of 4.0 (1.6–12.1) per 1000 persons and a median incidence of 15.2 per 100,000 individuals [1, 2]. The disease is characterized by positive and negative symptoms, including abnormalities in cognitive function and personality, such as language, thought, perception, emotion, and self-awareness. Despite the wide prevalence of SCZ and many decades of major progress, the underlying etiology of this disease remains elusive.

In contemporary practice, clinicians still diagnose clinical symptoms and evaluate progress and treatment responses based on clinical symptoms. The development of objective tests for the diagnosis of SCZ or monitoring of drug reactions is essential to providing early intervention for patients, which is beneficial for improving prognosis. In addition, several studies have shown that gene expression causes changes in different tissues of patients with SCZ [3–8]. The cystine/glutamate antiporter system x_c_^−^ is a potential target for the treatment of SCZ, which releases glutamate into synapses and thus increases glutaminergic neurotransmission [9, 10]. A human study also showed that the peripheral expression of mRNA of the two subunits of system x_c_^−^, solute carrier 3A2(SLC3A2) and solute carrier 7A11(SLC7A11), was lower in SCZ than healthy individuals [11]. Since the detection of mRNA in peripheral whole blood is accessible and non-invasive, the pattern of gene expression serves as a potential biomarker for SCZ diagnosis and therapeutic monitoring [12].

The *RELN* gene is located on chromosome 7q22, and it encodes the protein reelin, a large extracellular matrix glycoprotein that plays a key role in brain development from neuronal migration to dendritic spine formation and synaptic transmission [13–15]. Moreover, it may be involved in memory formation, neurotransmission, and synaptic plasticity [16, 17], as well as SCZ [18]. The earliest direct evidence was based on the postmortem studies of patients with SCZ, which revealed that *RELN* mRNA was reduced up to 50% in several regions of the brain [19, 20]. Additionally, *RELN* had been shown to be expressed in organ systems, inter alia in human blood, liver, pancreas, breast and intestines [21–26]. A recent study determined that the patients with SCZ had higher level of *RELN* gene methylation compared to healthy controls, leading to a subsequent 25-fold decrease in *RELN* expression in the methylated group [27]. The role of *RELN* was associated with SCZ in an earlier large genome-wide association study (GWAS), which found that the polymorphism rs7341475 accounted for a 1.4-fold increase in the risk of the disease in women [28]. Moreover, this gene variant was associated with working memory, episodic memory, and executive functioning in the nuclear families of one member with SCZ [29]. Subsequently, a growing number of studies have reported that a number of single nucleotide polymorphisms (SNPs) in the *RELN* gene were associated with the pathogenesis and/or severity of clinical symptoms of SCZ [30–37]. Hence, based on the low level of *RELN* in SCZ patients and the relationship between its genetic variation and SCZ, *RELN* may play a pathogenic role in SCZ [38]. This view was further supported by heterozygous reelin mouse model. Although the mice had fewer neuroanatomical defects, they had cognitive abnormalities of some common psychotic disorders [39].

In studies on SCZ, the healthy controls were not treated with antipsychotic drugs, whereas patients with SCZ were often treated with these drugs. It is important to determine whether changes in *RELN* gene expression in these patients are the result of long-term antipsychotic treatment or due to etiological characteristics of SCZ. Suzuki et al. reported that the level of the RELN receptor VLDLR in the peripheral blood lymphocytes of patients with SCZ was decreased. After 6 months of antipsychotic treatment, the gene expression increased [40]. Another study showed that protracted treatment with olanzapine resulted in the upregulation of *RELN* expression in the frontal cortex of rats [41, 42]. However, a previous postmortem study showed no correlation between the levels of *RELN* mRNA and reelin protein and lifetime doses of antipsychotics drugs [20]. Overall, the regulation of *RELN* in patients with SCZ requires broader research including other typical or atypical antipsychotic treatments.

In this study, we investigated the expression of *RELN* mRNA in whole blood before and after antipsychotic medication in patients with SCZ to explore the therapeutic value of *RELN* as a biomarker for SCZ.

## Methods and methods

### Subjects

All participants were unrelated and of the ethnic Han group from the Jiangsu Province of China. The SCZ patients were recruited from the Wuxi Mental Health Center and independently diagnosed by at least two experienced psychiatrists according to the Diagnosis and Statistical Manual of Mental Disorders, 4th ed. (DSM-IV).

A total of 30 patients in the acute stage of SCZ (16 women and 14 men; mean age = 35.87 years, SD = 10.21) were chosen for this study and included 5 first-episode, 18 drug-naive SCZ patients or recurrent, 7 drug-free SCZ patients with self-withdrawn antipsychotic drugs at least 1 month before enrollment (Table 1). All patients participating in the study were treated with one of the oral second generation or atypical antipsychotics (SGA) and received continuous drug therapy for 12 weeks after baseline assessments. The psychopathology of all patients was assessed through an oral case history interview and psychiatric interview using the Positive and Negative Syndrome Scale (PANSS) [43] at baseline and 12 weeks. Among the 30 patients, 20 were treated with risperidone, 6 with olanzapine, 2 with clozapine, 1 with quetiapine, and 1 with aripiprazole. All patients were administered one of the five SGAs once daily, starting with an initial dose and increasing to a curative dose over the subsequent 2–4 weeks. All patients demonstrated clinical improvement as evidenced by more than a 25% reduction in the PANSS score. A total of 48 healthy controls (HCs) (31 women and 17 men; mean age = 31.67 years, SD = 6.75) without a history of mental health disorders or neurological diseases were recruited from local communities by advertisement (Table 1).

**Table 1:** Demographic characteristics of the study participants

	Control	SCZ	*P* value
Sample size	48	30	
Age (mean ± SD)	31.67 ± 6.75	35.87 ± 10.21	0.066
Gender (Men: woman)	17:31	14:16	0.323

Abbreviations: *Control* Healthy control, *SCZ* Untreated SCZ patients

There were no significant differences in gender and age between the SCZ and HC groups. The Ethics Committees of the Wuxi Mental Health Center approved this study, and the study was conducted according to the principles of the Declaration of Helsinki 1975. Written informed consent was obtained from all participants prior to participation in the study.

### Analysis of *RELN* expression by the real-time quantitative PCR (RT-qPCR)

Whole-blood samples were collected in PAXgene blood RNA tubes (PreAnalytiX QIAGEN/BD, Hombrechtikon, Switzerland) and total RNA was extracted from whole blood samples using PAXgene Blood miRNA Kit (PreAnalytiX QIAGEN/BD, Hombrechtikon, Switzerland) according to the manufacturer’s instructions. Reverse transcription was performed using 200 ng of total RNA and the High Capacity RNA-to-cDNA Kit (Thermo Fisher Scientific, Waltham, MA, USA). Real-time quantitative PCR was performed using the primers for *RELN,* namely, CATGGTTGCAAGTGTGACCC (forward) and AAACCAGGGCCTTACCACTG (reverse) and a SYBR®Select Master Mix (Thermo Fisher Scientific, Waltham, MA, USA). The reactions were performed in triplicate for each of the three independent samples using the ViiA 7 Real-Time PCR System (Thermo Fisher Scientific, Waltham, MA, USA) under the following conditions: 95 °C for 10 min and 40 cycles each of 95 °C for 10 s and 60 °C for 60 s. The relative expression level of *RELN* for each individual after normalization to *ACTB* was calculated using the comparative Ct (2^−ΔΔCt^) method [44]. The primers of *ACTB* were AAGTCCCTCACCCTCCCAAAAG (forward) and AAGCAATGCTGTCACCTTCCC (reverse).

### Statistical analysis

The Statistical Package for the Social Sciences (SPSS) software version 21.0 (SPSS Inc., Chicago, IL, USA) for statistical analyses. The Student’s t-test was used to compare age between the SCZ group and control subjects. Gender differences were measured using Pearson’s chi-square test. PANSS scores before and after treatment were compared using the paired t-test.

The differences of *RELN* mRNA levels between the SCZ group and the healthy control group were analyzed for normality with the Shapiro-Wilk test followed by Mann-Whitney U test, and the changes in *RELN* expression between the SCZ group before and after treatment with antipsychotic medication were analyzed using paired Wilcoxon signed-rank test. All of the *P* values less than 0.05 were considered statistically significant. Spearman’s correlation analysis was used to analyze the relationship between the *RELN* mRNA levels and the PANSS scores in SCZ patients before and after treatment.

## Results

**Fig. 1:**
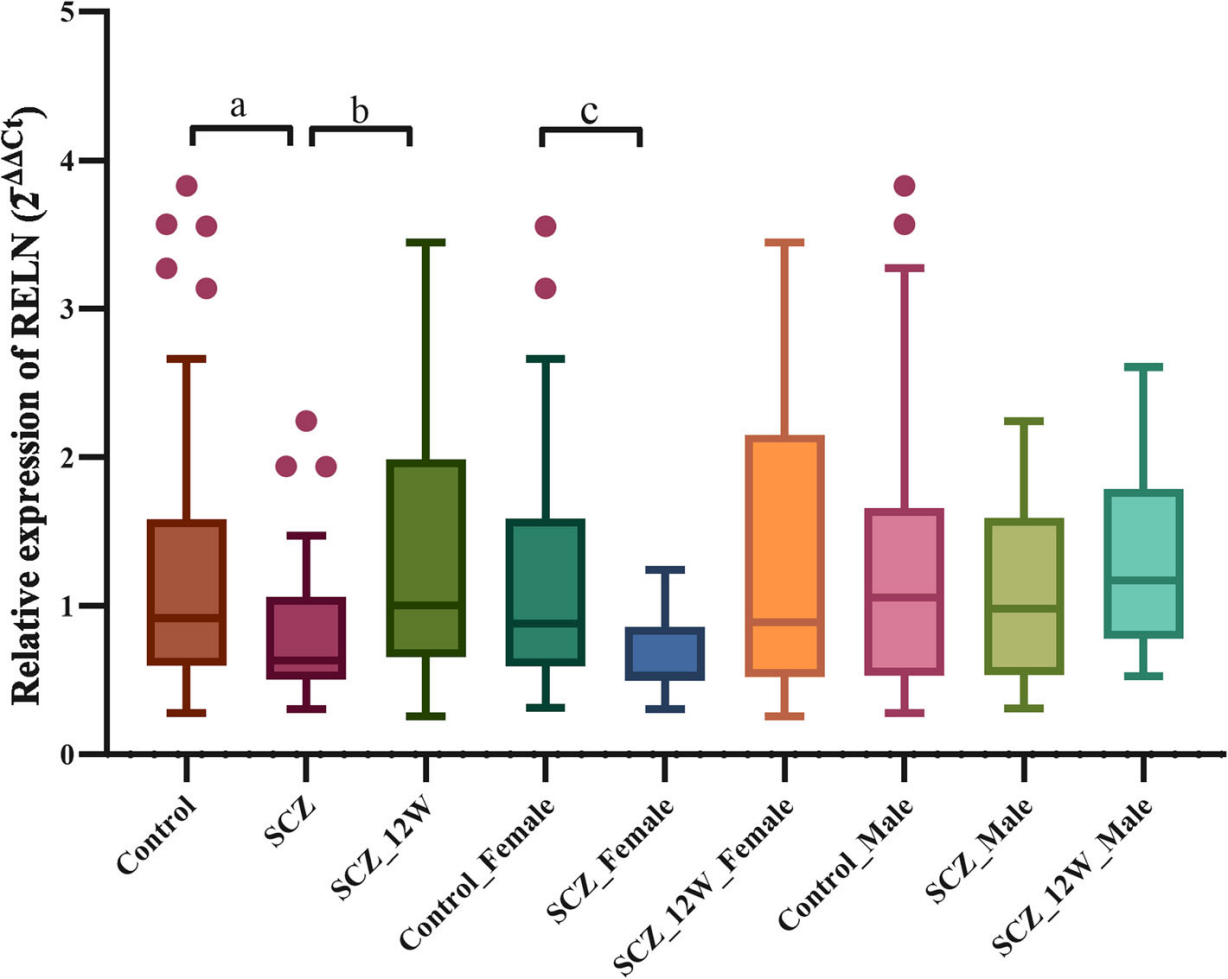
A Tukey’s “box-plot” of the expression levels of *RELN* in controls and SCZ patients before and after treatment (2^−ΔΔCt^). a: The expression of *RELN* mRNA in the whole blood of control subjects was higher than that in SCZ patients (Mann-Whitney U test); b: The expression of *RELN* mRNA in the whole blood of SCZ_12w patients was higher than that in SCZ patients (paired Wilcoxon signed-rank test); c: In female subgroup, the expression of *RELN* mRNA in the whole blood of control subjects was higher than that in SCZ patients (Mann-Whitney U test); Control: healthy control; SCZ: untreated SCZ patients; SCZ_12w: SCZ patients after 12 weeks of antipsychotic treatment. The box plots display the median and interquartile range (25th to 75th percentiles), and the whiskers outside the box extend to the highest and lowest value within 1.5 times the interquartile range. Points outside the whiskers are outliers

The expression levels of *RELN* mRNA in each subject were validated in the RT-qPCR shown in Fig. 1. The expression levels of *RELN* mRNA in the whole blood of 30 SCZ patients was significantly lower than that of the 48 healthy controls (*P* = 0.0476, Table 2). After 12 weeks of antipsychotic treatment, the expression levels of *RELN* mRNA in the SCZ patients were significantly increased (*P* = 0.0384, Table 2). The analysis of the expression levels of *RELN* mRNA in specific subgroups found that the *RELN* mRNA expression level of untreated female SCZ patients was significantly lower than that of the healthy female control group (*P* = 0.0025, Table 2).

**Table 2:** Comparison of *RELN* expression levels in controls, and SCZ patients before and after treatment (2^−ΔΔCt^)

	*RELN*	*P*
**Total**		
Control (48)	1.25 ± 0.13	
SCZ (30)	0.86 ± 0.09	**0.0476** ^**a**^
SCZ_12W (30)	1.32 ± 0.15	**0.0384** ^**b**^
**Female**		
Control (31)	1.20 ± 0.14	
SCZ (16)	0.64 ± 0.07	**0.0025** ^**a**^
SCZ_12W (16)	1.33 ± 0.24	0.0507 ^b^
**Male**		
Control (17)	1.35 ± 0.28	
SCZ (14)	1.10 ± 0.16	0.9844 ^a^
SCZ_12W (14)	1.32 ± 0.17	0.3575 ^b^

Abbreviations: *Control* Healthy control, *SCZ* Untreated schizophrenia patients, *SCZ_12w* Schizophrenia patients after 12 weeks of antipsychotic treatment; ^a^: comparisons between the untreated schizophrenia patients and healthy control (Mann-Whitney U test); ^b^: comparisons between the schizophrenia patients after 12 weeks of antipsychotic treatment and untreated schizophrenia patients (paired Wilcoxon signed-rank test); Bold figures indicate *P* < 0.05

As shown in Table 3, we still have distinguished one specific drug from the whole class of antipsychotic as *RELN* mRNA-induced dysregulation. After 12 weeks of olanzapine treatment, *RELN* mRNA expression level in 6 SCZ patients was significantly increased (*P* = 0.0313).

**Table 3:** Comparison of *RELN* expression levels in SCZ patients before and after treatment with different antipsychotics (2^−ΔΔCt^)

*RELN* (n)	SCZ	SCZ_12W	*P*
Risperidone (20)	0.94 ± 0.13	1.20 ± 0.15	0.2943
Olanzapine (6)	0.61 ± 0.09	1.85 ± 0.40	**0.0313**
Others (4)	0.80 ± 0.18	1.16 ± 0.57	> 0.9999

Abbreviations: Others: included 2 SCZ with clozapine, 1 SCZ with quetiapine, and 1 SCZ with aripiprazole. *SCZ* Untreated schizophrenia patients, *SCZ_12w* Schizophrenia patients after 12 weeks of antipsychotic treatment; Bold figures indicate *P* < 0.05

As shown in Table 4, in the SCZ patients before and after treatment, no significant relationship was found between the *RELN* mRNA levels and the positive symptom points, negative points, general pathological symptom points, or the total PANSS scores. In addition, we have observed no significant correlations between variation in *RELN* mRNA expression and the reduction rate of total PANSS scores (*r* = − 0.09, *p* = 0.638).

**Table 4:** Correlation analysis between *RELN* mRNA levels and PANSS scores in SCZ patients before and after treatment (*r* value)

PANSS	*RELN* mRNA	
	SCZ (*n* = 30)	SCZ_12W (*n* = 30)
Total PANSS scores	−0.021	− 0.196
Positive symptom points	−0.114	− 0.177
Negative symptom points	−0.041	− 0.174
General pathological symptom points	0.131	−0.246

Abbreviations: *SCZ* Untreated schizophrenia patients, *SCZ_12w* Schizophrenia patients after 12 weeks of antipsychotic treatment; All *P* values > 0.05

## Discussion

In this study, we found decreased expression of *RELN* mRNA in the whole blood of the untreated SCZ patients. This finding is consistent with previous studies in which *RELN* gene expression was down-regulated in the brain and peripheral blood of SCZ patients [19, 20, 27]. In addition, the study revealed that the *RELN* mRNA expression levels were significantly upregulated in SCZ patients after 12 weeks of antipsychotic treatment. Fatemi and his colleagues reached a similar conclusion in the frontal cortex of rats [41, 42]. Meanwhile, a similar conclusion was reached in the study of *RELN*-related genes in SCZ patients [40]. We still have taken into account the difference in drug treatment, and have distinguished olanzapine from the whole class of antipsychotic as *RELN* mRNA-induced dysregulation. Hence, the up-regulation of *RELN* mRNA expression was concurrent with the improvement in symptoms in SCZ patients after treatment, suggesting that the changes in *RELN* mRNA expression were attributed to the antipsychotic treatment.

The main aim of the extensive literature studies is to identify potential biomarkers that play an important role in clinical practice supporting the diagnostic and therapeutic of SCZ [45]. These studies are important for all medical and mental illnesses. However, there are still no reliable biomarkers to support the diagnosis of SCZ that mainly depends on clinical observation. Thus, the identification of biomarkers of SCZ could be ideally incorporated into diagnostic criteria and potentially used to evaluate symptom improvement. Although these potential biomarkers are still in their infancy, it would be interesting to search for potential biomarkers through blood-based or brain imaging approaches. For instance, G72, functioning as a D-amino acid oxidase (DAAO) activator (DAOA), plays an important role in dysregulation of the glutamate system, and has been found to be a potential biomarker with excellent sensitivity and specificity [45]. Lin et al. demonstrated that the plasma G72 protein levels were significantly increased in both medicated and drug-free patients with SCZ than in healthy controls [46] and the finding has been independently verified [47].

It is worth explicitly noting that the main limitation of this study was the limited sample size, which may reduce the statistical power to compare the level of *RELN* mRNA expression between different groups. Furthermore, we only measured *RELN* mRNA expression in peripheral blood; therefore, extrapolating these results to the brain should be treated with caution. These findings will need to be confirmed in future studies that involve a larger sample size. We have compared the expression of *RELN* mRNA in SCZ patients before and after antipsychotic treatment but did not compare expression of *RELN* mRNA in healthy controls before and after a 12-week time window. Therefore, one limitation of this study was to assess the *RELN* levels only once in healthy control group.

## Conclusions

Taken together, these results demonstrate that the expression of *RELN* is down-regulated in the peripheral blood of untreated SCZ patients, whereas its expression level is significantly up-regulated after 12 weeks of antipsychotic treatment. These findings suggest that changes in *RELN* expression are associated with antipsychotic treatment and may provide new clues for understanding the pathogenesis and treatment of SCZ.

## Data Availability

The datasets used and/or analysed during the current study are available from the corresponding author on reasonable request.

## Abbreviations

SCZ: Schizophrenia
GWAS: Genome-wide association study
SNPs: Single nucleotide polymorphisms
DSM-IV: Diagnosis and Statistical Manual of Mental Disorders, 4th ed.
SGA: The oral second generation or atypical antipsychotics
PANSS: Positive and Negative Syndrome Scale
HCs: Healthy controls
RT-qPCR: Real-time quantitative PCR
2^−ΔΔCt^: Comparative Ct
SPSS: The Statistical Package for the Social Sciences.
